# The exploration of N6-deoxyadenosine methylation in mammalian genomes

**DOI:** 10.1007/s13238-021-00866-3

**Published:** 2021-08-17

**Authors:** Xuwen Li, Zijian Zhang, Xinlong Luo, Jacob Schrier, Andrew D. Yang, Tao P. Wu

**Affiliations:** 1 Department of Molecular and Human Genetics, Baylor College of Medicine, Houston, TX 77030 USA; 2 Medical Scientist Training Program, Baylor College of Medicine, Houston, TX 77030 USA; 3 Huffington Center on Aging, Baylor College of Medicine, Houston, TX 77030 USA; 4 Dan L Duncan Comprehensive Cancer Center, Baylor College of Medicine, Houston, TX 77030 USA

**Keywords:** DNA N^6^-methyladenine (6mA), mammalian DNA modification, non-canonical mammalian DNA methylation

## Abstract

N^6^-methyladenine (N^6^-mA, m^6^dA, or 6mA), a prevalent DNA modification in prokaryotes, has recently been identified in higher eukaryotes, including mammals. Although 6mA has been well-studied in prokaryotes, the function and regulatory mechanism of 6mA in eukaryotes are still poorly understood. Recent studies indicate that 6mA can serve as an epigenetic mark and play critical roles in various biological processes, from transposable-element suppression to environmental stress response. Here, we review the significant advances in methodology for 6mA detection and major progress in understanding the regulation and function of this non-canonical DNA methylation in eukaryotes, predominantly mammals.

## INTRODUCTION

DNA methylation is a biological process by which the methyl group is covalently added to the DNA base. DNA methylation has been shown to play essential roles in numerous biological processes and human diseases, including gene expression regulation, chromatin organization, tumorigenesis, and neurodegeneration (Smith and Meissner 2013; Schübeler 2015; Luo et al. 2018a). Although several forms of DNA methylation, such as N^4^-methylcytosine (4mC), 5-methylcytosine (5mC), and N^6^-methyladenine (6mA), have been observed in prokaryotes and unicellular eukaryotes (Gorovsky et al. 1973; Bromberg et al. 1982; Sánchez-Romero et al. 2015), 5mC and its oxidation derivatives (5-hydroxymethylcytosine/5hmC, 5-formylcytosine/5fC, and 5-carboxylcytosine/5caC) were considered as the only forms of DNA epigenetic modifications in mammals (Li and Zhang 2014). After decades of exploration, recent studies have identified the presence of DNA 6mA in multiple species of eukaryotes, including mammals (Fu et al. 2015; Greer et al. 2015; Zhang et al. 2015; Wu et al. 2016). In prokaryotes, one of the most well-known functions of 6mA is to help the host recognize exogenous DNA and to protect endogenous DNA from restriction endonuclease (REase) cleavage (Marinus and Morris 1973, 1974). While the existence and biological function of 6mA in mammals have yet to be fully determined, several studies have provided convincing evidence for the presence of functional 6mA in the mammalian genomes (Wu et al. 2016; Xie et al. 2018; Hao et al. 2020; Zhang et al. 2020a; Li et al. 2020b). In this review, we will discuss recent advances in novel approaches for DNA 6mA detection in the eukaryotic genomes, the current understanding of the regulatory pathways involved in the deposition, recognition, and removal of 6mA, and the recent progress in understanding the biological function of 6mA in eukaryotes, predominantly mammals.

## BRIEF INTRODUCTION OF 6mA IN PROKARYOTES

The 6mA was first discovered in *Bacterium coli* by Dunn et al. in the 1950s (Dunn and Smith 1955, 1958). Subsequent studies revealed 6mA as a widespread presence throughout bacterial genomic DNAs (Vanyushin et al. 1968). DNA 6mA was shown to play a critical role in the restriction-modification (R-M) system (Boyer 1971; Smith et al. 1972; Marinus and Morris 1973), in which unmodified foreign DNA can be recognized and cleaved by REase, whereas the host genome is methylated and protected by its 6mA methyltransferase (MTase). However, these studies also revealed that only a small portion of 6mA is involved in R-M systems in *E*. *coli*, and the function for the majority of 6mA in *E*. *coli* remained elusive until the discovery of *dam* MTase. Marinus et al. observed an 84% reduction of 6mA levels in an *E*. *coli* strain with *dam-3* mutation, indicating *dam-3* as the gene that encodes the MTase responsible for a major portion of N^6^-adenine methylation in *E*. *coli* (Marinus and Morris 1974). Further investigation revealed that the *dam-3* mutant strain is more sensitive to UV irradiation and mitomycin C treatment. DNA isolated from *dam-3* mutant strains contains more single-stranded breaks, suggesting the protective effect of 6mA on DNA strand breaks(Marinus and Morris 1974). 6mA has also been shown to play critical roles in DNA replication initiation (Lu et al. 1994), DNA mismatch repair (Kramer et al. 1984), and gene expression regulation (Sternberg 1985; Robbins-Manke et al. 2005). Further studies are required to investigate whether these 6mA functions observed in prokaryotes are conserved in eukaryotes.

## THE DISCOVERY OF 6mA IN EUKARYOTES

In addition to prokaryotes, 6mA has been also identified as one of the primary DNA modifications in many unicellular eukaryotes for decades, such as *Tetrahymena thermophila* and *Tetrahymena pyriformis* (Gorovsky et al. 1973; Bromberg et al. 1982). Yet, the identification of 6mA in metazoan had been unsuccessful until recently due to the limitation of detection approaches and lack of specific model systems. With the significant advances in ultra-high-performance liquid chromatography coupled with triple-quadrupole tandem mass spectrometry (UHPLC-MS/MS) and third-generation single-molecule real-time sequencing (SMRT-seq), 6mA was detected in *Chlamydomonas* (Fu et al. 2015), *C*. *elegans* (Greer et al. 2015), and *Drosophila* (Zhang et al. 2015)*.* In 2016, the presence of 6mA, for the first time, was reported in mammalian genomes including mouse embryonic stem cells (mESCs) (Wu et al. 2016) and mouse kidney (Koziol et al. 2016). Later on, 6mA was reported in mouse brain (Yao et al. 2017; Li et al. 2019b), pig embryo (Liu et al. 2016), and the human genome (Xie et al. 2018; Xiao et al. 2018) (Table 1).

**Table 1:** The 6mA profile in different eukaryotic species

Organism	Tissue	Abundance (ppm) ^a^	Distribution	Function	Motif	Reference
*Chlamydomonas*	-	4,000~6,000	Transcription start sites (TSS)	Nucleosome positioning, transcriptional initiation	ApT	(Fu et al. 2015)
*C*. *elegans*	Whole worms	100~4,000	Evenly distributed	-	AGAA/GAGG	(Greer et al. 2015)
*Drosophila*	Embryos/Ovary/ Brain	10~700	Repetitive elements	Transposon activation	-	(Zhang et al. 2015)
	Brain	~26	Polycomb-binding Sites	Transcriptional repression	-	(Yao et al. 2018)
*X*. *laevis*	Testes	~0.9	Excluded from coding regions	-	ApG	(Koziol et al. 2016)
Zebrafish	Embryos/Germ cells	20~1000	Repetitive elements	-	CACACACA/CCTAGC/CAGCAG	(Liu et al. 2016)
Mouse	ESCs	~7	LINE1 transposons	Suppress transcription of L1 and surrounding genes	AGAA/GAAATA	(Wu et al. 2016)
	ESCs	0.4~0.8	-	Maybe correlated to DNA repair (NHEJ pathway)	-	(Liu et al. 2021)
	Brain	6.6~25.5	Repetitive elements	Suppress LINE expression	AC/CA	(Yao et al. 2017)
	Brain	(1~4) ^b^	-	May related to response of ﻿neurotoxic environmental pollutant	-	(Fernandes et al. 2021)
	Kidney	-	Excluded from coding regions	-	-	(Koziol et al. 2016)
	TS-like cells	~50	SIDD regions	Prevent SATB1–DNA binding, control euchromatin boundaries	-	(Li et al. 2020b)
Pig	Embryos/Germ cells	150~1,700	-	-	-	(Liu et al. 2016)
Human	Blood	~510	Exons and mtDNA	Promote transcription	[G/C]AGG[C/T]	(Xiao et al. 2018)
	Glioblastoma	~1000	Heterochromatic regions	Suppress transcription	TGGATGGATGGA/GAATGGAATGGA	(Xie et al. 2018)
	HepG2	~0.3	mtDNA	Prevent TFAM binding and suppress transcription	CTTATC	(Hao et al. 2020)

^a^ppm: parts per million dA

^b^ng 6mA per mg cerebellum tissue

## THE METHODS TO DETECT 6mA IN EUKARYOTES

In addition to UHPLC-MS/MS and SMRT-seq mentioned above, several other detection methods, such as antibody-based DNA immunoblotting, immunofluorescence staining, and sequencing, have been utilized to detect, locate, and quantify 6mA in multicellular eukaryotes. However, 6mA’s abundance was shown to be extremely low in most mammalian tissues or cell lines, making the detection and evaluation of mammalian 6mA a challenging endeavor. Here, we summarize the current methods used in detecting 6mA in eukaryotes and emerging approaches that may be adopted to further investigate the genomic distribution of 6mA (Table 2).

**Table 2:** Summary of the methods in detecting 6mA in eukaryotes

Methods	Application	Advantages	Disadvantages	Antibody dependent	References
UPLC-MS/MS	Abundance quantification	Ultra-high sensitivity	No sequence information, high operational cost	No	(Huang et al. 2015; Liu et al. 2017)
Immunoblotting	Abundance quantification	Easy to apply	Semi-quantitative	Yes	(Stott 1989)
Immunofluorescence	Subcellular localization	Multiple staining; heterogeneity detection	Semi-quantitative	Yes	(Im et al. 2019)
6mA-DIP-seq	Genomic localization	Whole-genome mapping	Low resolution	Yes	(Greer et al. 2015; Zhang et al. 2015; Wu et al. 2016)
6mA-CLIP-exo-seq	Genomic localization	Higher-resolution compared to DIP-seq	More complicated workflow	Yes	(Fu et al. 2015)
DA-6mA-seq	Genomic localization	Single base resolution	Motif dependent	No	(Luo et al. 2016)
SMRT sequencing	Genomic localization	Single base resolution	High false-positive rate when 6mA level is ultra-low	No	(Flusberg et al. 2010)
Oxford Nanopore sequencing	Genomic localization	Single base resolution	High false-positive rate	No	(Rand et al. 2017)
Ag^+^ mediated replication	Oligonucleotides	High specificity	Only applicable to known 6mA sites	No	(Hong et al. 2016)
6mA covalent functionalization	Genomic localization	Antibody independent	Low labeling efficiency (~10%)	No	(Nappi et al. 2020)
Nitrite sequencing	Oligonucleotides	Single base resolution, antibody independent	Can only detect 6mA in a high-abundance context	No	(Mahdavi-Amiri et al. 2021)

### Mass spectrometry-based methods

Mass spectrometry, a powerful analytical technique, has been widely used to accurately quantify known compounds of biological samples. The ultra-high sensitivity (on the order of 0.00001%) makes mass spectrometry practical for detecting the low abundant 6mA in mammalian DNA. Besides, liquid chromatography (LC) or high-performance liquid chromatography (HPLC) can be used to separate DNA 6mA from RNA N6-methyladenosine (m^6^A), other nucleotides, and other DNA modifications. Thus, liquid chromatography coupled with mass spectrometry (LC-MS/MS) provides an ideal method to reliably detect the low abundant 6mA in the mammalian genome (Huang et al. 2015; Liu et al. 2017).

Before conducting LC-MS/MS to determine the abundance of 6mA, genomic DNA extracted from cells or tissues need to be enzymatically hydrolyzed to mononucleotides. It was suggested that the commercial enzymes used to hydrolyze DNA can be contaminated by bacterial nucleotides, resulting in an overestimation of 6mA level in eukaryotic genomes by LC-MS/MS (O’Brown et al. 2019). However, a more recent LC-MS/MS experiment has determined that the suspected contamination is below 0.1 ppm or detection limits, while the 6mA ranges from 7 in embryonic stem cells to 120 ppm in developing trophoblast stem cells in murine (Li et al. 2020b). Thus, with proper controls, LC-MS/MS remains a reliable approach to quantify the abundance of 6mA. Recently, one method developed by Dr. Hailin Wang’s lab introduced a metabolic labeling approach for accurate detection of 6mA and eliminating most false positive signals (Liu et al. 2017).

Although the mass spectrometry-based methods can accurately quantify the low abundance of 6mA, it requires protocol development and optimization to achieve adequate specificity and sensitivity, which is often challenging and time-consuming (Wetzel and Limbach 2016). For instance, studies reported that 6mA in mESCs and human pluripotent stem cells (hPSCs) is below the detection limit of their LC-MS/MS methods (Ratel et al. 2006; Schiffers et al. 2017; Abakir et al. 2020). By contrast, Li et al. reported a high 6mA level (>1000 ppm) in human embryonic stem cells (hESCs), human mesenchymal stem cells (hMSCs), and human vascular smooth muscle cells (hVSMCs) in their LC-MS/MS experiments (Li et al. 2020a). Additionally, a lower level of 6mA was detected by dot blotting in mESCs cultured under traditional 2i condition (ERK and GSK3b inhibitors, the culture condition used by Schiffers et al.) than those cultured in serum and LIF condition (Li et al. 2020b). In a separate study, Liu et al. reported that a similar level of 6mA was detected by LC-MS/MS in mESCs cultured in 2i condition or serum and LIF condition (Liu et al. 2021), and that 6mA level in their mESCs is around 0.4–0.8 ppm, which is ten-fold lower than previously reported result (Wu et al. 2016). Recently, Fernandes et al. reported that 6mA is a ubiquitous eukaryotic epigenetic modification that is put in place during embryogenesis and fetal development (Fernandes et al. 2021). Therefore, more studies are required to determine not only 6mA abundance but also how 6mA is regulated to confer cellular adaptation in different culture conditions. It is also worth noting that mass spectrometry-based methods cannot detect genomic localization of 6mA since the DNA sequence information was lost during hydrolysis step, which makes it challenging to identify possible contamination. Therefore, it is important to apply other independent methods to cross validate the results from mass spectrometry-based methods.

### Antibody-based detection methods

Compared to LC-MS/MS, antibody-based methods are generally less sensitive but much easier to apply. Therefore, antibody-based DNA immunoblotting (Stott 1989) and immunofluorescence staining (Im et al. 2019) are more routinely used for the initial evaluation of 6mA levels. Because the current 6mA antibody recognizes both RNA m^6^A and DNA 6mA, it is essential to remove the RNAs prior to the application of antibody-based methods.

Another advantage of the antibody-based method is that the antibody can be used in DNA immunoprecipitation followed by next-generation sequencing (DIP-seq) to specifically enrich methylated DNA fragments and generate genomic profiling of 6mA (Fu et al. 2015; Greer et al. 2015; Zhang et al. 2015; Wu et al. 2016). Similar to histone modification chromatin immunoprecipitation (ChIP-seq), 6mA DIP-seq can only detect 6mA sites at a resolution of 100–500 bp. To further increase the resolution of DIP-seq, Fu et al. developed the 6mA-CLIP-exo technique by employing photo-crosslinking followed by exonuclease digestion to achieve a much higher resolution than traditional DIP-seq in *Chlamydomonas* (Fu et al. 2015). Similar methods were subsequently applied to human genomic and mitochondrial DNA (Koh et al. 2018; Hao et al. 2020).

While antibody-based methods are easy to apply and versatile in detecting global level, subcellular localization, as well as genomic localization of 6mA, the specificity of immunoprecipitation needs to be established in one’s experimental system as any other antibody-based epigenetic detection method. The global 6mA level has been found as a critical parameter for conventional 6mA DIP-seq. A careful﻿ “Spike-in” experiments with standard oligonucleotides determined that the threshold for 6mA DIP is around 10–15 ppm of 6mA in the input samples in order to achieve above 10-fold enrichment over the background signal (Wu et al. 2016). Thus, it is not surprising that majority of 6mA DIP signals from human T-cells and other cell lines with ultra-low level of 6mA were found that might be nonspecific (Douvlataniotis et al. 2020). A recent study demonstrated that their denaturing DNA 6mA IP experiment can effectively enrich 6mA modified oligo but not m^6^A modified DNA-RNA hybrid (Li et al. 2020b). Furthermore, the 6mA DIP-seq in developing trophoblast stem cells resulted in similar peak pattern against input, IGG, or WGA controls, demonstrating the high specificity of the 6mA DIP signal when 6mA abundance is above the threshold (Li et al. 2020b). Nevertheless, well-controlled experiments, especially the inclusion of proper negative controls, are essential to study 6mA with antibody-dependent methods.

### Antibody-independent detection methods

To eliminate the concern of the potential artifacts from 6mA antibody, it is crucial for the field to develop antibody independent detection methods to cross-validate the results from antibody-based methods. Here, we summarize the recently developed antibody independent detection methods that may improve our understanding of 6mA in mammals.

The first type of antibody-independent 6mA detection method is based on digestion with methylation-sensitive restriction enzymes, which specifically cleave methylated or unmethylated DNA. For 6mA, DpnII (only digests unmethylated GATC), CviAII (only digests unmethylated CATG), and DpnI (only digests methylated GATC and CATC, preferentially fully methylated) have been used to detect 6mA at single-base resolution (Fu et al. 2015; Luo et al. 2016, 2018b). However, this method is limited by the motifs of restriction enzymes, so it is mostly applied in unicellular eukaryotes. As 6mA DIP-seq and SMRT-seq failed to detect these motifs in the mammalian genomes (Wu et al. 2016; Xie et al. 2018; Zhu et al. 2018; Xiao et al. 2018), methylation-sensitive restriction enzyme methods are unlikely to be widely used to detect genomic localization of 6mA in mammals.

Another antibody-independent method is to directly sequence native DNA using third-generation single-molecule sequencing. Pacific Bioscience’s single molecular real-time sequencing (SMRT-seq) detects the 6mA by taking advantage of the fact that DNA polymerase kinetics change while replicating modified or unmodified bases (Flusberg et al. 2010). SMRT-seq has been successfully applied to detect 6mA in plants and mammals (Wu et al. 2016; Liang et al. 2018; Zhou et al. 2018; Zhu et al. 2018; Xiao et al. 2018). However, the false-positive signal of SMRT-seq in detecting 6mA was also discussed (Zhu et al. 2018; Douvlataniotis et al. 2020). Therefore, 6mA enrichment, high sequencing coverage are required to reliably detect 6mA using SMRT-seq. It was recently reported that another platform of single-molecule sequencing, Oxford nanopore sequencing, can also detect DNA modifications at single-base resolution. Nanopore sequencing detects DNA methylation by examining the current difference generated by methylated and unmethylated DNA molecules when going through the sequencing pore (Rand et al. 2017). Computational tools have been developed to detect 6mA in prokaryotes and *Chlamydomonas* from nanopore sequencing data (Stoiber et al. 2016; Rand et al. 2017; Liu et al. 2019; McIntyre et al. 2019; Ni et al. 2019). However, no such tool was developed for metazoan genomes, largely due to the lack of 6mA data at single-base resolution from an accurate method, like bisulfite sequencing for 5mC. While 6mA detection from nanopore sequencing is still in the early stage, rapidly developing computational methods in methylation calling and base-calling makes nanopore sequencing a deserving method in detecting 6mA in mammals.

Additionally, researchers are also actively developing chemical methods for 6mA detection. Hong et al. discovered that Ag^+^ causes primer extension termination at 6mA sites but not unmethylated A sites, which can be used to selectively detect 6mA (Hong et al. 2016). However, the inability to exponentially amplify DNA fragments made this method difficult for the genome-wide mapping of 6mA. Alternatively, Nappi et al. reported a method to selectively label 6mA by photo-conjugation followed by biotin-ligation (Nappi et al. 2020). This label of 6mA can be subsequentially pulled down and cleaved, leaving the enriched 6mA fragment that can be quantified by qPCR or sequencing. Nappi et al. further demonstrated that the enrichment by this method is comparable to 6mA antibody-based immunoprecipitation. Furthermore, Mahdavi-Amiri et al. reported nitrite sequencing, a method to detect DNA 6mA and RNA m^6^A at single-base resolution (Mahdavi-Amiri et al. 2021). Nitrite sequencing utilizes the sodium nitrite and acetic acid to diazotize and deaminate unmethylated adenine, while methylated adenine cannot complete the deamination. Therefore, unmethylated adenine will be converted to guanine after PCR, while methylated adenine will remain as adenine. By calculating the relative A to G mutation ratio at each base, 6mA can be distinguished and quantified at single-base resolution. However, this method requires a high fraction of methylated adenine. Thus, it is challenging to directly apply this method to eukaryotic genome because 6mA methylation fraction is likely to be low. On the other hand, since 6mA methylation fraction in prokaryotic genome is high enough, this method can be an alternative method for SMRT sequencing to detect 6mA at single-base resolution.

Although DNA 6mA detection in the mammalian genomes is challenging due to the low abundance in most adult tissues and cell lines, novel detection methods are being rapidly developed. A detection method with high sensitivity, high specificity, and single-base resolution is a bottleneck in 6mA’s study. Such a method will greatly move the entire field forward.

## THE REGULATION of 6mA

The deposition and active removal of epigenetic modifications are mostly catalyzed by enzymes. For example, the canonical methylation on cytosine is catalyzed by DNA methyltransferases (MTase) enzymes (DNMT1, DNMT3A, and DNMT3B), and the demethylation process of 5mC can be either passive (through cell division) or active (catalyzed by ten-eleven translocation enzymes (TETs)) (Moore et al. 2013). Several studies reported the dynamic regulation of 6mA and the putative machinery responsible for the deposition, recognition, and removal of mammalian 6mA (Wu et al. 2016; Xie et al. 2018; Xiao et al. 2018; Kweon et al. 2019; Hao et al. 2020; Li et al. 2020b). The challenging questions in the field are whether the methylation of N6-deoxyadenosine in the mammals is catalyzed by MTase, and subsequently, by which MTase.

### Putative methyltransferases of 6mA in mammals

Although significant effort has been put into the search for 6mA MTases in mammals, the exploration turned out to be challenging due to the low abundance of 6mA and the lack of functional model systems to work on. Nevertheless, several groups have identified putative MTases of mammalian 6mA. Several studies have suggested that N-6 adenine-specific DNA methyltransferase 1 (N6AMT1), the mammalian MTase similar to the prokaryotic DNA 6mA MTase (M. TaqI), may mediate the methylation of N6-deoxyadenosine in human liver cancer cells and mouse neurons (Xiao et al. 2018; Li et al. 2019b; Sheng et al. 2021). However, Xie et al. did not observe such activities of N6AMT1 in human glioblastoma (Xie et al. 2018). Additionally, recent structural and biochemical evidence indicated that N6AMT1 could not bind to DNA to catalyze the methylation of 6mA (Li et al. 2019a).

**Figure 1:**
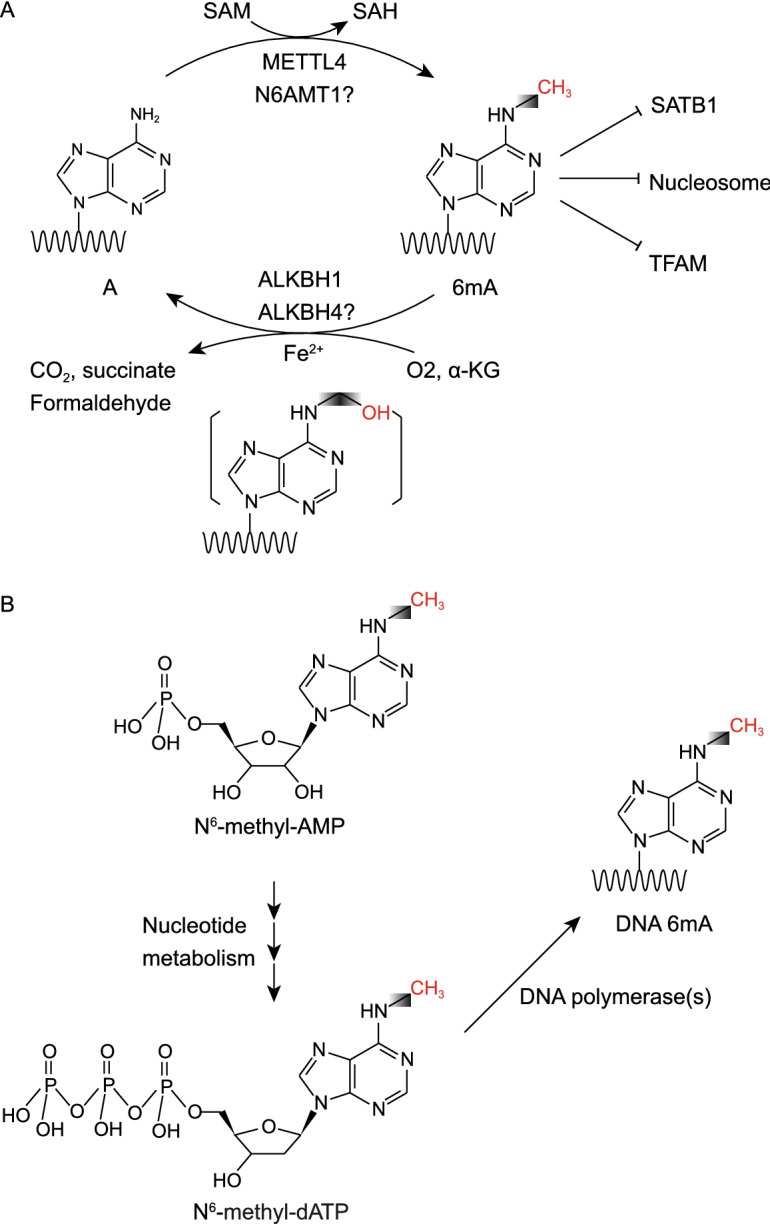
**Regulation of DNA 6mA in the mammalian genome**. (A) Methylation and demethylation pathway and biochemical function of 6mA in the mammalian DNA. Methyltransferase METTL4 or N6AMT1 catalyze the transfer of methyl group from the methyl donor S-adenosylmethionine (SAM) to unmethylated A, leading to a 6mA site and S-adenosylhomocysteine (SAH). 6mA can be oxidatively demethylated by ALKBH1 and ALKBH4 using oxygen, alpha-ketoglutarate (α-KG), and ferrous ion (Fe^2+^), leading to an unstable intermediate 6hmA and rapidly decays to adenine and formaldehyde. 6mA has been shown to directly prevent nucleosome, SATB1 and TFAM from binding to DNA. (B) 6mA can also be directly incorporated into DNA during DNA replication or DNA damage repair. N^6^-methyl-dATP can be formed from the N^6^-methyl-AMP, degraded from methylated RNA, through nucleotide metabolism pathway

Another putative MTase of DNA 6mA is METTL4. METTL4 is a homologous protein of MTA-70 family eukaryotic MTases in mammals. Its homologs in *C*. *elegans* (DAMT-1) and *T*. *thermophila* (TAMT-1) were identified as the MTase of 6mA (Greer et al. 2015; Luo et al. 2018b). Kweon et al. reported that METTL4 catalyzes 6mA deposition in genomic DNA of human embryonic kidney cells and mESCs by overexpressing and knocking out METTL4 (Kweon et al. 2019). Consistently, METTL4 has been reported as a 6mA MTase during the differentiation of murine 3T3-L1 cells (Zhang et al. 2020b). Furthermore, Hao et al. demonstrated that METTL4 could mediate 6mA methylation of mitochondrial DNA (mtDNA) in HepG2 cells and is capable of catalyzing mtDNA 6mA methylation under *in vitro* biochemical conditions (Hao et al. 2020). Of note, exposing HepG2 cells to hypoxia conditions resulted in an upregulation of METTL4 and a corresponding increase in mtDNA 6mA level (Hao et al. 2020). It is worth noting that Woodcock et al. demonstrated that the METTL3-METTL14 complex, a well-known mRNA m^6^A MTase complex, can also mediate *in vitro* DNA 6mA methylation on GGACT motif in both single-strand DNA (ssDNA) and unpaired double-strand DNA (dsDNA) oligonucleotides (Woodcock et al. 2019). However, 6mA on such a motif has not been reported in genomic profiling studies (Koziol et al. 2016; Wu et al. 2016; Yao et al. 2017; Xie et al. 2018; Xiao et al. 2018). Further investigations are needed to demonstrate whether the METTL3-METTL14 complex can catalyze DNA methylation in mammalian cells. Nevertheless, METTL4 is currently a promising candidate for the mammalian 6mA MTase (Fig. 1A). The *in vitro* biochemical evidence of METTL4 as the MTase for 6mA in genomic DNA is still lacking, and experiments in multiple different systems are required to validate the DNA MTase activity of METTL4. At the same time, it is crucial to search for additional proteins that may function as DNA 6mA MTase. Moreover, studies to examine the effect of various substrates on the methylation reaction should also be critical.

### Other possible sources of physiological 6mA in mammals

The low abundance of 6mA in mammalian DNA and the lack of definitive DNA 6mA MTase mammalian setting raise concerns that 6mA may be mis-incorporated by DNA polymerases during DNA replication and DNA damage repair rather than by direct DNA methylation via MTases. A previous report from Charles et al. showed that 6mA could be detected in the genomic DNA of C2C12 cells treated with free N^6^-Methyl-2’-deoxyadenosine using HPLC–MS/MS but not in untreated cells, indicating that 6mA can be incorporated into the mammalian genome (Charles et al. 2004). Musheev et al. showed that while DNA 5mC are directly mediated by DNA MTase, DNA 6mA may instead originates from ribo-N^6^-methyladenosine degraded from m^6^A modified RNA and not from direct methylation in 3T3 and C2C12 cells (Fig. 1B) (Musheev et al. 2020). In a separate study, Liu et al. reported that DNA 6mA accumulates in the G_1_ phase and can be incorporated into genomic DNA in mESCs by template-independent polymerase λ, the major polymerase participating in the non-homologous end joining (NHEJ) DNA repair pathway (Liu et al. 2021). Consistent with the study from Musheev et al., Liu et al. also observed increased genomic 6mA level after the treatment of exogenous ribo-N^6^-methyladenosine, while failing to detect direct methylation activity by possible 6mA MTases. These results also suggest that 6mA may be incorporated primarily during DNA repair rather than during DNA replication in mESCs. Overall, these studies proposed that the physiological basal level of 6mA in normal mammalian cell lines may originate from misincorporation by DNA polymerases. However, Musheev et al. showed that the endogenous level of soluble 6mA nucleoside is extremely low or undetectable and *in vitro* PCR results from Liu et al. revealed that N^6^-methyl-dATP is not the preferred substrate by high fidelity DNA polymerase (Musheev et al. 2020; Liu et al. 2021). Furthermore, it is unclear whether this misincorporation mechanism may account for the high dynamic characteristics of 6mA when responding to stress like hypoxia or in pathological conditions (Xie et al. 2018). It is possible that the 6mA methyltransferase is only active at specific conditions or in specific niche. Therefore, more evidence is required to either demonstrate or rule out the direct methylation of 6mA by DNA MTase.

### The recognition of 6mA

To fully understand the biological function of 6mA in eukaryotes, it is important to identify proteins that recognize (“read”) 6mA. In prokaryotes like *E*. *coli*, MutH and SeqA have been reported to recognize and bind the hemi-methylated GATC sites to promote DNA mismatch repair and prevent premature DNA replication (Kramer et al. 1984; Lu et al. 1994). He et al. reported that a Fox-family transcription factor Jumu can bind to 6mA modified DNA and negatively regulate the expression of Zelda, a zygotic early *Drosophila* activator (He et al. 2019). This regulation further controls the proper zygotic genome activation (ZGA), indicating an important role of 6mA in *Drosophila* early embryogenesis. Koh et al. reported that mitochondrial single-stranded DNA binding protein 1 (SSBP1) can be recruited by 6mA in HEK293T mitochondria, perhaps due to the dsDNA destabilizing property of 6mA (Koh et al. 2018). However, the downstream function of the 6mA-mediated recruitment of mitochondrial SSBP1 is still unclear. A study by Hao et al. has revealed that 6mA in mtDNA can disrupt the DNA binding of mitochondrial transcription factor A (TFAM) to suppress mtDNA transcription, further demonstrated that 6mA plays an important role in the human mitochondrial genome (Fig. 1A). Nevertheless, the proteins that recognize 6mA in mammalian nuclear DNA remain elusive. Surprisingly, Li et al. demonstrated that 6mA antagonizes special AT-rich sequence binding protein 1 (SATB1), a crucial chromatin organizer, in stress-induced DNA double helix destabilization (SIDD) regions during the transition from mESCs to trophoblast stem-like cells (TSC) (Fig. 1A). The enrichment of 6mA in SIDD regions, in which double-stranded DNA is frequently unpaired, is consistent with structural result that the demethylase ALKBH1 prefers unpairing DNA as substrates (Zhang et al. 2020a; Tian et al. 2020). This antagonizing (anti-reader) effect of 6mA on SATB1 binding is supported by both *in vitro* binding assay and chromatin profiling. Further investigation revealed that this antagonizing effect of 6mA on SATB1 modulates the cell fate transition from mESCs to TSCs (Li et al. 2020b). This study provides an alternative mechanism of 6mA function by preventing proteins from binding to DNA in mammalian cells. It is still possible that there are other unidentified proteins that can bind to 6mA modified DNA in mammalian cells. Further exploration is required to fully understand the recognition machinery or antagonizing effector of 6mA and the functional roles in mammalian settings.

### The demethylases of 6mA

Unlike the determination of 6mA methyltransferases in mammals, the search for 6mA demethylase has yield significant progress since its report in mESCs. Several studies utilizing genetic and biochemical methods have demonstrated that ALKBH1 can function as the DNA 6mA demethylase in the mammalian genome. Structural evidence also supported that ALKBH1 can bind unpairing DNA and catalyze the demethylation of 6mA (Zhang et al. 2020a; Tian et al. 2020). ALKBH1 is a homolog of alpha-ketoglutarate-dependent dioxygenase (AlkB), which is a bacterial protein responsible for the repair of alkylated DNA and RNA by oxidative demethylation (Falnes et al. 2002). ALKBH1 catalyzes 6mA demethylation by hydroxylating the methyl group to a hydroxymethyl group (Zhang et al. 2020a). This unstable hydroxymethyl group can be spontaneously released as formaldehyde, leading to direct demethylation in contrast to the multi-step 5mC demethylation (Fig. 1A). These enzymatic and structural experiments revealed that ALKBH1 prefers bubbled or bulged DNA instead of conventional single or double-stranded DNA, possibly due to the lack of autonomous base-flipping activity (Zhang et al. 2020a). Consistently, several studies indicate that 6mA can modify DNA fine structure by destabilizing the DNA double-strand helix (Diekmann 1987; Koh et al. 2018). Further studies are required to understand more of ALKBH1 in mammals.

In addition to ALKBH1, ALKBH4 has also been reported to function as a demethylase of DNA 6mA (Kweon et al. 2019). ALKBH4 is orthologous to DMAD and NMAD-1, the 6mA demethylase identified in *D*. *melanogaster* and *C*. *elegans*, respectively (Greer et al. 2015; Zhang et al. 2015; Yao et al. 2018). However, Kweon et al. showed that ALKBH4 primarily demethylate short double-strand oligonucleotides rather than single-strand oligonucleotides, while 6mA is predominantly enriched in unpairing regions in mESCs (Li et al. 2020b). Yet so far, ALKBH4’s role as 6mA demethylase has not been reported by other groups. Thus, more studies are required to confirm whether ALKBH4 can demethylate DNA 6mA in multiple systems.

## THE BIOLOGICAL FUNCTION OF 6mA IN MAMMALS

While the critical function of 6mA in replication, transcription, and R-M system are well-recognized in prokaryotes, the physiological and pathological roles of 6mA in the mammals are still elusive. Particularly, it is still disputable whether the rare 6mA in the mammalian genome could play an important biological function. Nevertheless, various emerging evidence supports that 6mA can serve as an important modification that responds to different stresses, such as hypoxia (Xie et al. 2018; Hao et al. 2020). In addition, 6mA may be crucial in early development and some pathological conditions, such as cancer and neurological disorders (Yao et al. 2017; Xie et al. 2018; Li et al. 2019b, 2020b).

### 6mA in early embryonic development

6mA in the mammalian genome was first identified in mESCs and results in significant transcriptional repression at its deposition sites, especially on young long interspersed nuclear element-1 (LINE1) retrotransposons (Wu et al. 2016). The accumulation of 6mA in mESCs results in imbalanced cell fate decisions during *in vitro* embryoid body differentiation by regulating the expression of key developmental genes such as Nanog, Cdx2, Lefty1&2, and Foxa2 (Wu et al. 2016). Consistently, 6mA was found to be dynamically regulated during early embryogenesis in zebrafish and pigs (Liu et al. 2016). However, whether 6mA plays a direct role in early embryo development was unclear. A recent study suggested that 6mA plays a key role in placenta development (Li et al. 2020b). Concretely, Li et al. found that a high level of 6mA is present in cells transitioning from mESCs to mouse trophoblast stem (TS)-like cells. Genomic profiling revealed that 6mA is enriched at regions of SIDD, and 6mA deposition was found to function at euchromatin boundaries to restrict the spread of euchromatin by preventing SATB1 chromatin association. Additionally, 6mA-SATB1 antagonism was reported to be essential for trophoblast development in both the cell line and mouse model. This work is the first to demonstrate that 6mA may play an essential role in murine early development. More research is needed to investigate whether 6mA has a similar role in other organisms.

### 6mA in brain

In addition to its function in early development, many efforts have been made to investigate the role of 6mA in the mammalian brain. Yao and colleagues discovered that the 6mA level in the prefrontal cortex (PFC) was increased from 6.6 ppm to 25.5 ppm in mice exposed to chronic stress (Yao et al. 2017). Consistent with the genomic distribution of 6mA in mESCs, genomic profiling indicated that increased 6mA by chronic stress were enriched at repetitive elements like LINE1. Additionally, transcriptomic profiling showed 6mA negatively correlates with the expression of LINE retrotransposons and a group of neuronal genes. Similarly, another study demonstrated 6mA accumulated in primary cortical neurons after neuronal activity was induced *in vitro* (Li et al. 2019b). The study also investigated 6mA levels in neurons that can be selectively activated by fear extinction learning in the infralimbic prefrontal cortex (ILPFC) of adult mice. However, DpnI-seq identified a surprisingly large number of 6mA sites (more than two million sites), which was far more abundant than the estimated level by the DIP-seq method in PFC (Yao et al. 2017). It is possible that such a difference was observed due to different contexts, stress types, or detection methods. It would be interesting to see whether this high level of 6mA can be verified by LC-MS/MS. Overall, these studies suggest that 6mA has an important function in the mammalian brain, especially PFC. An important question is whether 6mA plays a role in pathological conditions, such as neurological diseases.

### 6mA in cancer

DNA methylation is not only essential for early mammalian development but also crucial for tumorigenesis (Kulis and Esteller 2010). While the critical role of 5mC in regulating transcription during cancer development is well recognized, how other types of DNA modifications like 6mA may contribute to tumorigenesis remains elusive. Xie et al. reported that 6mA is highly upregulated and a potential therapeutic target in human glioblastoma (Xie et al. 2018). Genomic profiling showed that 6mA is co-localized with heterochromatic histone modifications, predominantly H3K9me3 in glioblastoma stem cells (GSCs). To investigate the function of 6mA in glioblastoma, Xie et al. knocked-down ALKBH1, the demethylase of 6mA, and observed an increase in 6mA and H3K9me3 level as well as an inhibition in GSC growth. Further analysis showed that ALKBH1-sensitive 6mA are significantly enriched in and regulating key oncogenic pathways, including the hypoxia response pathway. Consistently, 6mA was reported to be elevated in human esophageal squamous cell carcinoma (Chen et al. 2020). In contrast, studies have reported a decrease of DNA 6mA level in primary gastric, liver, and lung cancers (Liang et al. 2016; Xiao et al. 2018). It is possible that the expression, regulation, and function of 6mA vary in different types of cancer. Further investigations are required to elucidate the regulatory mechanism and functional role of 6mA in cancer.

### 6mA in mitochondria

The endosymbiotic hypothesis for the origin of mitochondria suggests that mitochondria are descended from specialized prokaryotes, a concept supported by the similarity of mitochondrial DNA (mtDNA) and bacterial genome (Gray 2012). Whether mtDNA preserves high levels of 6mA like bacteria and the function of 6mA in mtDNA have attracted much attention in the field. Several publications detected a higher level of 6mA in mtDNA than in nuclear DNA via different detection methods (Koh et al. 2018; Xiao et al. 2018; Hao et al. 2020). Koh et al. first systematically investigated mitochondrial 6mA in HEK293T cells. Their UHPLC-MS/MS data showed a significantly higher level of 6mA in mtDNA (~18 ppm) compared to genomic DNA (<1 ppm). Functionally, Koh et al. observed a decrease in mitochondrial oxidative phosphorylation after knocking-out ALKBH1, the demethylase of 6mA. Similarly, Hao et al. investigated the level of mitochondrial 6mA in multiple cell or tissue types, including HepG2 cells, 143B cells, MDA-MB-231 cells, mouse primary fibroblast cells, testes, and spleens. Their UHPLC-MS/MS results showed significant enrichment of 6mA in mtDNA in all samples tested, especially in HepG2 cells (up to 400 ppm in mtDNA). Additionally, they identified the co-localization of a potential MTase (METTL4) and mitochondria in HepG2 cells. Consistent with the previous study, Hao et al. observed an elevated protein level of the OXPHOS complex III component upon METTL4 knockdown. Furthermore, Hao et al. demonstrated 6mA suppresses mitochondria gene transcription by preventing TFAM from binding to DNA. Finally, Hao et al. found that hypoxia can further elevate 6mA in mtDNA, indicating mitochondrial 6mA may play an important role in regulating the hypoxia stress response. Together, these studies demonstrated a significant enrichment of 6mA in mtDNA compared to nuclear DNA, and that 6mA in mtDNA plays a crucial role in regulating mitochondria activity and responding to environmental stress like hypoxia. It will be interesting to test whether there is crosstalk between mitochondria 6mA and the nuclear epigenetic regulation.

### 6mA in DNA damage repair

While the role of 6mA in DNA mismatch repair is well-established in prokaryotes, it is still unclear whether this role of 6mA is conserved in eukaryotes. Zhang et al. have proposed an elegant model that DNA 6mA at nucleotide excision repair (NER) sites may prevent the misincorporation of 8-oxoguanine (8-oxoG), as the misincorporation of 8-oxoG into DNA opposite to unmethylated adenine will lead to a transversion mutation from T:A to G:C (Zhang et al. 2021). Thus, DNA 6mA may function as a protective DNA modification to prevent the transversion from T:A to G:C. One concern of this model is that majority of single-stranded adenine sites after nucleotide excision need to be methylated to efficiently prevent misincorporation of 8-oxoG. Given the replication error rate (~1 in 100, 000) and the average length of single-stranded DNA gap during NER (~25–30 nt), the abundance of 6mA need to be around 62.5 to 75 ppm to prevent the incorporation of 8-oxoG, which is at least one order of magnitude higher than previously reported basal level of 6mA in many cell lines (~0.1–7 ppm). Nevertheless, the model may hold true in more specific conditions, such as the repair of UV-induced DNA damage and double strand breaks.

**Figure 2:**
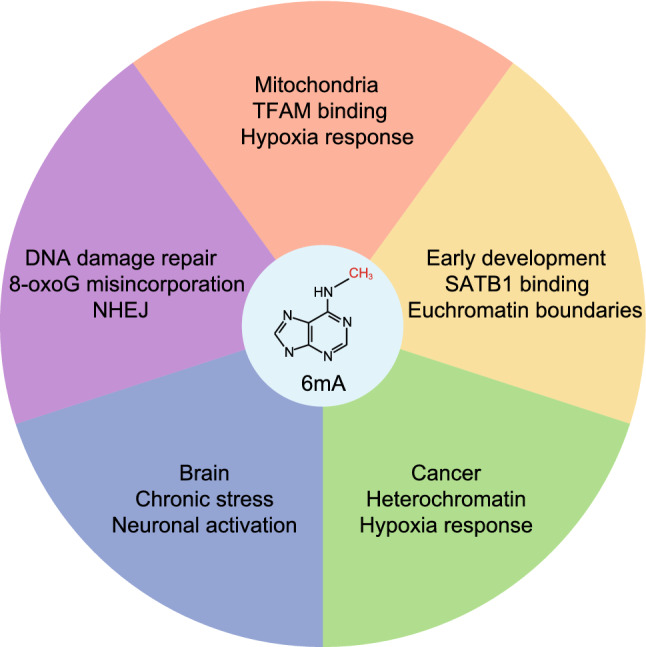
**The proposed function of 6mA in mammals**. 6mA has been reported to play important roles in mammals, such as mitochondrial activity, early development, tumorigenesis, brain function, and DNA damage repair

In another study, Liu et al. reported that 6mA is accumulated at the G_1_ phase and polymerase lambda (*pol λ*) can directly incorporate 6mA into DNA in mESCs, indicating that 6mA may also play a role in NHEJ pathway (Fig. 1B, 2) (Liu et al. 2021). However, the exact role of 6mA in the NHEJ pathway remains unknown. Further investigation is required to better understand the function of 6mA in DNA damage repair.

Collectively, although 6mA has been identified in various eukaryotic species, the functional research of 6mA in these species is still in the initial stage. Many of the studies showed that 6mA is more dynamically regulated, compared to 5mC, by different environmental stress, including hypoxia (Xie et al. 2018; Hao et al. 2020), chronic stress (Yao et al. 2017), extinction learning (Li et al. 2019b), and mitochondrial stress (Ma et al. 2019). These discoveries indicate that 6mA can serve as an epigenetic mark that quickly responds to different environmental stresses and protects cells from more severe consequences. Further studies are required to investigate the detailed mechanisms of how these environmental stresses induce the upregulation of 6mA. Moreover, these conditions should be utilized to help identify the possible methyltransferases or readers of 6mA in different functional models.

## CONCLUSION AND OUTLOOK

DNA 6mA has been identified in many eukaryotic species in recent years (Fu et al. 2015; Greer et al. 2015; Zhang et al. 2015; Wu et al. 2016; Xiao et al. 2018). Functionally, 6mA has been shown to play critical roles in regulating gene expression, retrotransposon suppression, stress response, chromatin organization, tumorigenesis, and early embryonic development (Wu et al. 2016; Yao et al. 2017; Xie et al. 2018; Li et al. 2020b). Research on the regulation of 6mA in the mammalian genome is still in the early stage. Whether the origin of 6mA in the mammalian genome are through DNA MTases like METTL4 or from misincorporation of methylated bases by DNA polymerases remains elusive (Kweon et al. 2019; Hao et al. 2020; Musheev et al. 2020; Liu et al. 2021). The removal of 6mA is mediated by demethylases like ALKBH1 and ALKBH4 (Wu et al. 2016; Kweon et al. 2019). While 6mA has been shown to prevent DNA binding of nucleosome and proteins (SATB1 and TFAM) (Luo et al. 2018b; Beh et al. 2019; Hao et al. 2020; Li et al. 2020b), the existence of “reader” proteins that specifically recognize and bind 6mA sites in mammals is still an open question. Thus, additional research into the mechanism pathways of deposition, removal, and recognition of 6mA in mammals is crucial to further understand its function. Particularly, deciphering the methyltransferase of 6mA in mammalian settings remains the major challenge.

Another major challenge for the research on 6mA is the lack of methods to efficiently map 6mA at single-base resolution via currently available strategies. We believe that with the advances in chemical and third-generation sequencing methods, there will soon be breakthroughs in the methodology of 6mA detection. These novel methods will greatly help researchers better characterize the function of 6mA in the mammals.

The last but not least, 6mA functional roles should be explored in the specific model systems and the right “niche”.
